# Neutrophils in the Pathogenesis of Rheumatoid Arthritis and Systemic Lupus Erythematosus: Same Foe Different M.O.

**DOI:** 10.3389/fimmu.2021.649693

**Published:** 2021-03-04

**Authors:** Michele Fresneda Alarcon, Zoe McLaren, Helen Louise Wright

**Affiliations:** ^1^Institute of Life Course and Medical Sciences, University of Liverpool, Liverpool, United Kingdom; ^2^Liverpool University Hospitals National Health Service (NHS) Foundation Trust, Liverpool, United Kingdom

**Keywords:** neutrophils, rheumatoid arthritis, systemic lupus erythematosus, NETs, low density granulocytes, immunometabolism

## Abstract

Dysregulated neutrophil activation contributes to the pathogenesis of autoimmune diseases including rheumatoid arthritis (RA) and systemic lupus erythematosus (SLE). Neutrophil-derived reactive oxygen species (ROS) and granule proteases are implicated in damage to and destruction of host tissues in both conditions (cartilage in RA, vascular tissue in SLE) and also in the pathogenic post-translational modification of DNA and proteins. Neutrophil-derived cytokines and chemokines regulate both the innate and adaptive immune responses in RA and SLE, and neutrophil extracellular traps (NETs) expose nuclear neoepitopes (citrullinated proteins in RA, double-stranded DNA and nuclear proteins in SLE) to the immune system, initiating the production of auto-antibodies (ACPA in RA, anti-dsDNA and anti-acetylated/methylated histones in SLE). Neutrophil apoptosis is dysregulated in both conditions: in RA, delayed apoptosis within synovial joints contributes to chronic inflammation, immune cell recruitment and prolonged release of proteolytic enzymes, whereas in SLE enhanced apoptosis leads to increased apoptotic burden associated with development of anti-nuclear auto-antibodies. An unbalanced energy metabolism in SLE and RA neutrophils contributes to the pathology of both diseases; increased hypoxia and glycolysis in RA drives neutrophil activation and NET production, whereas decreased redox capacity increases ROS-mediated damage in SLE. Neutrophil low-density granulocytes (LDGs), present in high numbers in the blood of both RA and SLE patients, have opposing phenotypes contributing to clinical manifestations of each disease. In this review we will describe the complex and contrasting phenotype of neutrophils and LDGs in RA and SLE and discuss their discrete roles in the pathogenesis of each condition. We will also review our current understanding of transcriptomic and metabolomic regulation of neutrophil phenotype in RA and SLE and discuss opportunities for therapeutic targeting of neutrophil activation in inflammatory auto-immune disease.

## Introduction

Systemic Lupus Erythematosus (SLE) is the archetypal autoimmune connective tissue disease, characterized by the production of multiple auto-antibodies [anti-nuclear antibodies (ANA), anti-double stranded DNA (dsDNA), anti-Sm/RNP, anti-Ro/La] and the consumption of complement (1, 2). It has the capacity to involve almost any organ system of the body resulting in protean and sometimes catastrophic consequences for patients. SLE disproportionately affects young women and is a condition with a broad spectrum of severity, ranging from mild joint involvement to life-threatening organ failure (3). Typical manifestations include systemic complaints, such as overwhelming and intrusive fatigue, brain fog, fever, swollen lymph nodes, mouth ulcers, chilblains, and weight loss (4). Rashes are common in SLE and may be transient or disfiguring; rashes are often triggered by sunlight and may be associated with hair loss and scarring. The characteristic malar flush of redness over the cheeks gives the name lupus (from wolf) and is likened to the appearance of a butterfly. Inflammation of joints, tendons, and muscle may cause arthritis, nodules, or contractures and give rise to disability and pain. Kidney disease including inflammation and immune complex deposition may occur which, if untreated, can lead to kidney failure and the need for dialysis and transplant (5, 6). Inflammation can develop in the pleural lining of the lungs, around the heart and may even affect the heart muscle and valves. SLE may also affect any part of the nervous system from brain and spinal cord to the peripheral nerves resulting in neurological problems, such as strokes, neuropathy, headache, visual loss, migraine, confusion, and acute psychosis (7). The disease may cause a fall in blood counts involving specific cell lines or indeed pancytopenia and may be associated with serious abnormalities of both clotting and bleeding. Repeated inflammatory insults, abnormal blood clotting and the consequence of treatment with high dose steroids and immunosuppression can also lead to chronic illness through damage accrual and increase the likelihood of infection, osteoporosis, premature ovarian failure, cardiovascular events such as atherosclerosis, stroke or heart attack, and malignancy. SLE is incurable and with modern treatment is still associated with an increased risk of mortality, and shortened life expectancy (3).

Rheumatoid arthritis (RA) is a chronic, autoimmune, systemic inflammatory condition associated typically with antibodies to rheumatoid factor (RF) and cyclic citrullinated peptides (anti-CCP or ACPA) (8). It is the commonest form of inflammatory arthritis and is characterized by inflammation of the tendon sheaths (tenosynovitis) and joint lining (synovitis) leading to growth of an inflammatory pannus which quickly erodes the joint cartilage and bone, causing recognizable deformities that were once commonplace in rheumatology clinics (9). Untreated or resistant to therapy, RA results in a symmetrical, deforming polyarthropathy. This leads to physical disability, progressive loss of function and as well as stiffness and pain. Extra-articular complications of the disease include interstitial lung disease, vasculitis, nodules, eye disease and an increased risk of cardiovascular disease, malignancy and osteoporosis (10, 11). Modern management is focused on a prompt diagnosis and early use of immunosuppressive treatments, including traditional and biologic disease-modifying anti-rheumatic drugs (DMARDs), with the aim of targeting disease remission. This has led to a reduction in the need for orthopedic surgery for patients with this form of arthritis, a move to out-patient based care as opposed to long hospitalizations, and a reduction in the systemic complications that can occur. However, RA still remains an incurable disease with treatments that rely on the long-term suppression of the immune system resulting in side effects and complications, including an increased risk of infection (9, 12).

Both RA and SLE are caused by a dysregulation of the innate and adaptive immune systems, including clonal expansion of auto-reactive lymphocytes, production of auto-antibodies and elevated production of multiple cytokines and other inflammatory mediators. Research into the underlying cause of both diseases focusses heavily on dysregulated T- and B-cell responses (9, 12, 13). However, it is inappropriately activated neutrophils that have the greatest potential to cause damage to local tissues, both due to their presence in high numbers at sites of inflammation and through release of their cytotoxic contents directly onto host tissues. Neutrophils are specialist cells of the innate immune system that normally play a major role in host defense against microorganisms through phagocytosis and generation of reactive oxygen species (ROS). Production of ROS within the phagosome occurs via the action of NADPH oxidase (NOX2) and myeloperoxidase (MPO) which, together with release of proteases from granules and vesicles into the phagosome, provide a defensive arsenal against a broad spectrum of microscopic pathogens. During infection, ROS and proteases may be released extracellularly causing local tissue damage at the site of infection (14). This damage is normally resolved by resident macrophages, which remove apoptotic neutrophils and damaged tissue as part of the normal process of inflammation resolution (15). Neutrophils are the most abundant leukocyte in humans, being produced by the bone marrow in huge numbers daily (estimated to be in the region of 5–10 × 10^10^ per day) (16, 17). Whilst the majority of neutrophils circulate in the blood (both free-flowing and marginated to the endothelial vessel walls), several populations of tissue neutrophils exist within healthy homeostasis, including within the lung, spleen and liver (18). These tissue neutrophils play a major role in surveillance and host defense, B-cell Ig-class switching, and phagocytosis of circulating bacteria, respectively. The liver is also a major site for efferocytosis and removal of neutrophils that have been involved in bacterial killing (18). Neutrophil release from the bone marrow, and subsequent homing back to bone marrow for efferocytosis at the end of their normal life span, is regulated by circadian expression of *CXCR2* (receptor for CXCL2), the central clock gene *BMAL1* and *CXCR4* (receptor for CXCL12) (19), with granule content and the ability to produce NETs being the highest in the morning, decreasing throughout the day to reach the lowest levels by mid-afternoon in human neutrophils (20).

Neutrophils contribute to inflammation and tissue damage in inflammatory disease, when they become inappropriately activated by cytokines, chemokines and auto-antibodies (14). Auto-immune neutrophils function in a multitude of ways to direct the inflammatory response, including release of proteases which damage host tissue and activate soluble proteins (21), secretion of cytokines and chemokines which direct both the innate and adaptive immune responses (22), shedding of receptors, such as the interleukin-6 receptor to initiate trans-signaling (23, 24), release of neutrophil extracellular traps (NETs) providing a source of auto-antigens (25), and production of ROS (8, 26). This review will discuss the dysregulation of neutrophil activation in RA and SLE, two auto-immune diseases characterized by aberrant neutrophil activation. We will highlight how uncontrolled neutrophil activation contributes to the development of auto-immunity and disease progression, and summarize how inappropriate neutrophil activation might be targeted with therapeutics.

## Apoptosis

Neutrophils are terminally differentiated cells which, in the absence of inflammation, circulate in the blood for around 24–48 h before returning to the bone marrow and undergoing apoptosis (27). Constitutive neutrophil apoptosis is regulated by Bcl-2 family proteins: anti-apoptotic MCL1 and A1/BFL1, and pro-apoptotic BAX, BAK, and BID. Loss of MCL1 and BFL1 causes BAX:BAK pore formation in the mitochondrial membrane, releasing cytochrome c which along with APAF1 forms the apoptosome, leading to cleavage of caspases initiating apoptosis (14, 27–29). Neutrophil apoptosis may be delayed during inflammation by cytokines (e.g., GM-CSF, TNFα), bacterial lipopolysaccharide (LPS) and leukotrienes (e.g., leukotriene B4), which extend the life-span of neutrophils through stabilization or up-regulation of MCL1 and/or BFL1 (30–35). Aging neutrophils can be identified in circulation by decreased expression of L-selectin (CD62L) and CXCR2, and increased expression of CXCR4, the receptor for CXCL12; it is the increased signaling via CXCL12:CXCR4 which enables aged neutrophils to home back to the bone marrow to undergo apoptosis and clearance (19). Neutrophil apoptosis may be induced through activation of death-receptor signaling pathways, such as FASL, TRAIL, and TNFα which directly activate caspase-8 and caspase-3, and induce degradation of BID and BAX leading to mitochondrial release of cytochrome c (28, 36). Intriguingly, TNFα may be pro- or anti-apoptotic *in vitro* depending upon the concentration in media, with low concentrations delaying apoptosis and higher concentrations promoting apoptosis (37). Whilst not completely understood, this effect is believed to be mediated via the two TNF receptors (TNFR1 and TNFR2), with TNFR1 signaling anti-apoptotic activation of NF-κB, and TNFR2 activating death receptor signaling (38, 39).

**Figure 1:**
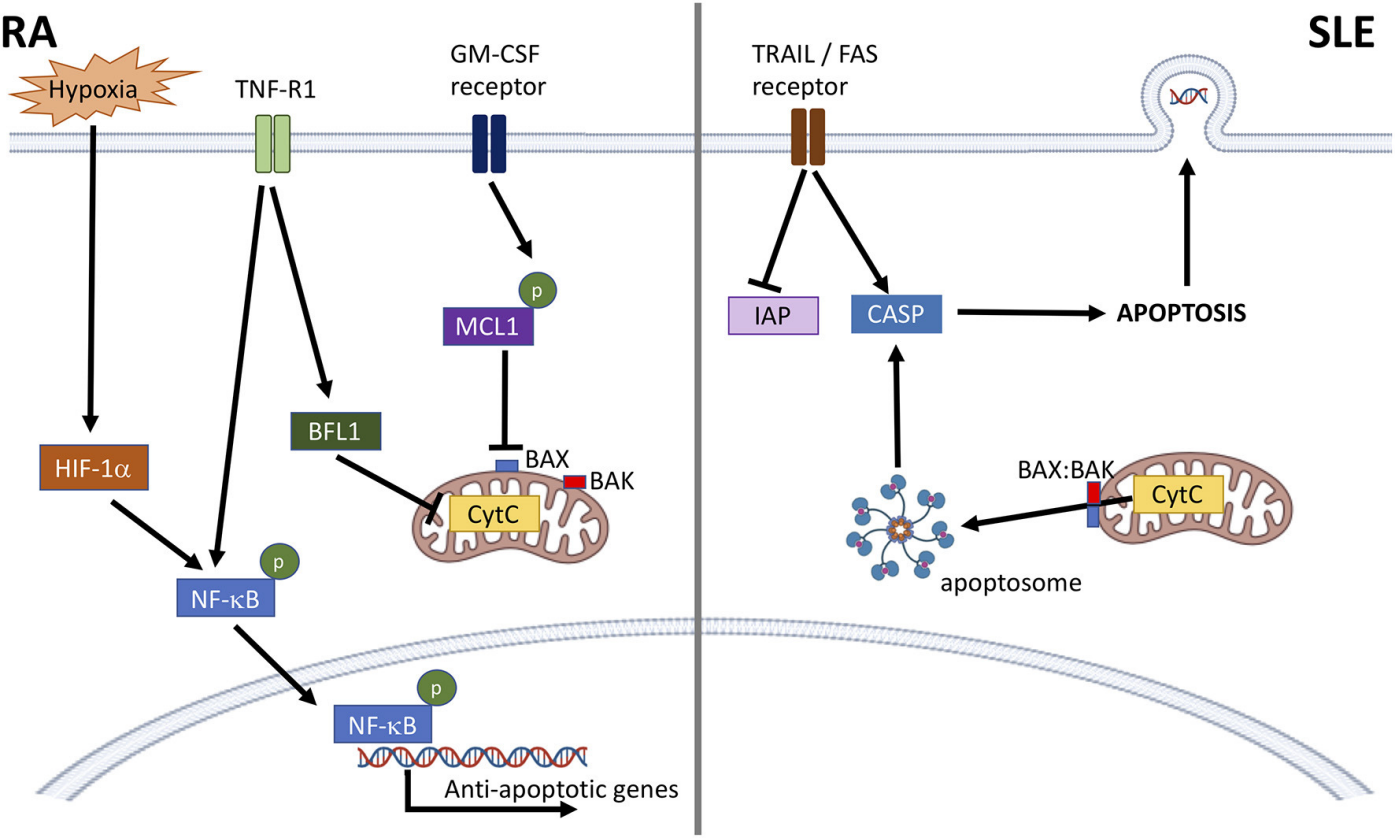
Dysregulation of neutrophil apoptosis in RA and SLE. In RA, anti-apoptotic factors, such as cytokines and hypoxia activate NF-κB and prevent mitochondrial cytochrome c (CytC) driven apoptosis through activation of BFL1, and stabilization of MCL1. This prevents BAX:BAK pore formation, CytC leakage and formation of the apoptosome. In SLE, activation of death receptors (e.g., TRAIL, FAS receptors) lowers levels of cellular inhibitors of apoptosis (IAP) and activates caspase-8 (CASP). BAX:BAK pore formation in the mitochondrial membrane releases CytC leading to apoptosome formation and activation of CASP-9 and CASP-3 leading to apoptosis. Nuclear antigens, including DNA, are also expressed at the plasma membrane.

In SLE, neutrophil apoptosis is enhanced (40–42) leading to increased apoptotic burden associated with development of anti-nuclear auto-antibodies (25, 43). A dysregulation between pro-apoptotic caspases and inhibitors of apoptosis (IAP1, IAP2, XIAP) may explain the enhanced apoptosis seen in SLE neutrophils *in vitro* (Figure 1) (41, 42, 44). Levels of pro-apoptotic TRAIL and FASL are also significantly higher in SLE serum, which can induce apoptosis in healthy neutrophils (41). Levels of GM-CSF are lower in SLE serum, and supplementation of SLE serum with physiological levels of GM-CSF can rescue the pro-apoptotic effect of SLE serum on healthy neutrophils (42). Incubation of apoptotic neutrophils with SLE PBMCs induces expression of interferon-alpha (IFNα) via a toll-like receptor (TLR)-dependent mechanism (45). SLE neutrophils also express nuclear antigens (dsDNA) at the plasma membrane, and this effect can be induced in healthy neutrophils incubated with SLE serum (46). As well as an increase in neutrophil apoptosis, defects in clearance of apoptotic cells by macrophages can also contribute to the accumulation of apoptotic debris in SLE (47).

Apoptosis is delayed in both blood and synovial fluid neutrophils from patients with RA (48, 49). The likely cause of this is inflammatory cytokines, such as GM-CSF, TNFα, IL1β, and interferons, elevated in both blood and synovial fluid (50), which have been demonstrated to delay neutrophil apoptosis in *in vitro* experiments (32–34, 37, 51–55). RA blood neutrophils express higher levels of MCL1, higher levels of phosphorylated NF-κB and lower levels of active caspase-9 compared to healthy controls (48). Delayed apoptosis in synovial fluid has also been attributed to other factors including lactoferrin and adenosine (56, 57). The hypoxic environment in RA synovial joints also plays a key role in delaying neutrophil apoptosis, via increased expression of MCL1 (Figure 1) (58). Hypoxia can also delay apoptosis via stabilization of hypoxia-inducible factor 1-alpha (HIF1-α) and activation of NF-κB (59), and regulates neutrophil retention at sites of inflammation, prolonging inflammation (60).

Microparticles (MPs) are small (0.2–2 μM) extracellular vesicles containing cargos of protein, lipids and nucleotides (e.g., RNA, miRNA) which are released from activated cells via budding and shedding of the cell membrane (61). MPs are distinct from other extracellular vesicles, i.e., exosomes and apoptotic bodies, by virtue of their size, cargo, membrane content, and mode of formation (61). MPs are taken up by neighboring cells through endocytosis, and their protein and RNA cargo can alter the recipient cell phenotype once released into the cytoplasm. They represent a novel form of paracellular communication and differ in composition depending upon the functional state of the originating cell. In SLE, MPs are annexin V^+^ and contain cell markers indicating both endothelial cell and neutrophil origins (62). SLE MPs activate plasmacytoid dendritic cells to produce a range of cytokines, including IFNα (62), and stimulate ROS production in autologous and healthy neutrophils (63). Acetylated histones within chromatin in MPs suggests the MPs are derived from apoptotic cells (62) and may be central to the stimulation of NOX2-independent NETs (64). The population of annexin V^+^/acetylated histone^+^ MPs appear to be specific to SLE and are not present in sera of healthy controls or individuals with RA (62). MPs released from RA neutrophils may have anti-inflammatory properties, activated through the pro-resolving protein Annexin-A1 (65, 66). Annexin-A1 is released from neutrophil granules following extravasation and is found in RA synovial fluid. This protein exerts its pro-resolving effect by promoting apoptosis and decreasing neutrophil:endothelial cell adhesion and extravasation (66, 67).

## ROS Mediated Tissue Damage

Neutrophils produce ROS via activation of the NADPH oxidase (NOX2) (68). NOX2 is a multi-component enzyme, which is assembled at the phagosomal and plasma membrane following cytokine priming (69, 70). Priming induces phosphorylation and mobilization of granular and cytosolic NOX2 components [p22^phox^ and gp91^phox^ which together comprise cytochrome b_558_, p40^phox^, p47^phox^, p67^phox^, and Rac (Rac-1 or Rac-2)] to the phagosomal and plasma membranes in readiness for phagosomal killing (70–73). Assembly of NOX2 at the plasma membrane leads to the release of ROS into the extracellular environment and is a major cause of damage to host tissue in RA and SLE. ROS production via NOX2 in primed neutrophils is triggered by activation of cell receptors [e.g., FcγR, complement receptors, f-Met-Leu-Phe (fMLP) receptor]. Activated NOX2 catalyses the reduction of oxygen to superoxide (O_2_^-^), an unstable oxygen radical which rapidly forms hydrogen peroxide (H_2_O_2_), the hydroxyl free radical (HO^•^) and/or peroxynitrite (${\text{NO}}_{3}^{-}$) depending on cellular conditions (68, 74). Hydrogen peroxide is the major substrate of myeloperoxidase (MPO), a neutrophil azurophilic granule enzyme implicated in the production of highly-reactive, secondary oxidants, such as hypochlorous, hypobromous, and hypothiocyanous acids which are potent anti-microbial agents that damage proteins, lipids and DNA (68, 75).

SLE neutrophils exhibit aberrant ROS production, with O_2_^-^, H_2_O_2_, and HO^•^ being produced more rapidly and in higher levels than healthy individuals (76, 77). DNA, protein and lipid markers of intracellular oxidative stress are increased in SLE neutrophils (76), including 8-hydroxyguanosine, an oxidized self-DNA which may function as a damage-associated molecular pattern (DAMP) (78) and which is present in NETs (79). Neutrophils from SLE patients with circulating immune complexes or cytotoxic antibodies produce the highest O_2_^-^ response to FcγR/complement receptor stimulation (80), and unstimulated *ex vivo* neutrophils from lupus nephritis patients have the highest levels of ROS production (77). SLE serum induces O_2_^-^ generation in healthy neutrophils, with O_2_^-^ production correlating positively with the presence of immune complexes and negatively with the concentration of complement (81). Many patients with SLE experience both flares and periods of inactive or quiet disease. Neutrophils from SLE patients in active flare often produce lower levels of ROS *in vitro* than those with inactive disease, possibly due to exhaustion of the neutrophils *in vivo* (76), or lower expression of Fcγ and complement receptors (82). As yet unidentified factors within SLE serum induce production of cytokines by human renal glomerular endothelial cells which promote neutrophil chemotaxis and adhesion (83). In patients with lupus nephritis, immune complexes become deposited within tissues in the kidney (6). This may arise through the binding of anti-nucleosomes and anti-C1q auto-antibodies to nucleosomes and C1q captured on the surface of glomerular endothelial cells by heparan sulfate (84). Neutrophils expressing FcγR2A adhere to immune complex deposits on the surface of glomerular capillaries, activating ROS production and release directly onto host tissue (85). This release of ROS is directly responsible for damage to glomeruli, including oxidation of DNA, lipids and proteins, and induction of apoptosis (86). Oxidized high-density lipoprotein (HDL), commonly found in SLE patients, is pro-inflammatory, driving production of IL-6 and TNF by macrophages, and lacking its normal, cardioprotective properties (87). A polymorphism in the gene for neutrophil cytosolic factor 1 (NCF1, rs201802880) in a sub-set of SLE patients is associated with lower ROS production by neutrophils (88). This polymorphism also increases expression of type 1 interferon-regulated genes (89), the importance of which will be discussed later in this review. Interestingly, the NCF1 (m1J) mutated mouse model of SLE has demonstrated a link between ROS deficiency and interferon-driven autoimmunity downstream of a deficient NOX2 complex (90).

**Figure 2:**
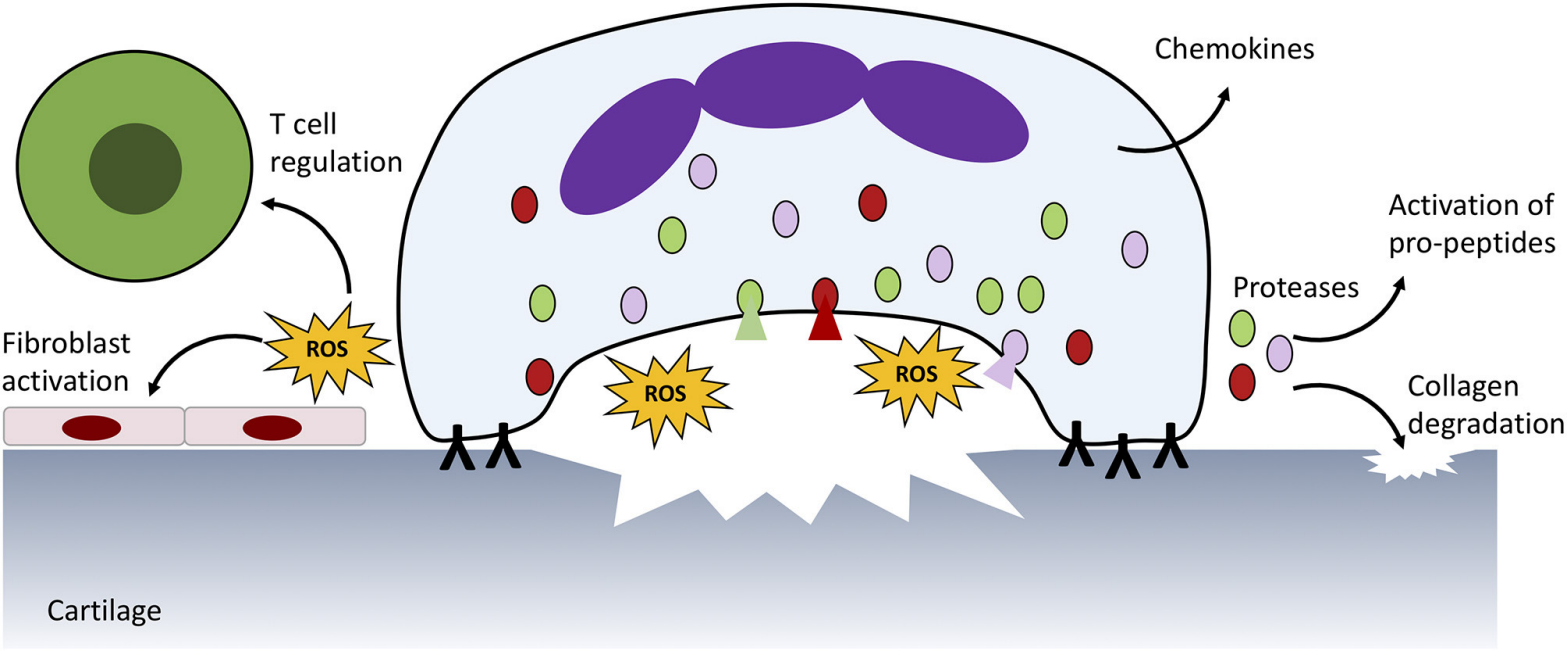
Neutrophil ROS production and protease release damages host tissue. In RA, immune complexes on the surface of the joint activate ROS production and protease release from granules (shaded red, green, and pink) which damages underlying cartilage and activates neighbouring immune cells and fibroblasts. Proteases also activate pro-peptides, such as cytokines produced by neutrophils and other infiltrating immune cells. A similar process is responsible for damage to blood vessels in SLE.

In RA, both blood and synovial fluid neutrophils have an increased capacity to produce ROS (91, 92). ROS production within RA blood leads to modifications of immunoglobulin G (IgG) which are associated with increased immunogenicity and production of rheumatoid factor immune complexes (93). Within the RA joint IgG complexes, both soluble and embedded within synovial tissue, activate further ROS production by neutrophils via activation of FcγR2a and FcγR3b (Figure 2) (94, 95), and trigger degranulation of proteolytic enzymes including elastase and cathepsin G (8, 26, 56, 96–98). When this occurs at the articular surface a microenvironment of concentrated ROS, proteases and cytotoxic factors is formed, damaging the underlying structures (8). As well as damaging collagen fibers within cartilage, neutrophil granule proteases cause proteolytic cleavage and activation of proteins (matrix metalloproteinases, pro-cytokines/chemokines) and cleavage of soluble receptors to initiate trans-signaling (such as the IL-6 receptor) (8, 21, 23, 99–102). Additionally, ROS production within the joint disrupts oxidative homeostasis and drives adaptive immune responses to the synovial environment (68). ROS-induced MAPK and NF-κB activation in synovial fibroblasts activates production of pro-inflammatory prostaglandins by cyclooxygenase (COX)-2 (103). ROS also exert profound effects on the local T cell population, regulating differentiation, apoptosis and cytokine production (104, 105).

## Neutrophil Extracellular Traps

Neutrophil extracellular traps (NETs) are mesh like DNA structures decorated with histones, MPO and other antimicrobial proteins expelled from neutrophils in response to infectious or inflammatory stimuli (106). They are an alternative defense mechanism by which neutrophils trap and possibly kill microbes (106, 107). Whilst the early events that signal NET production rather than phagocytosis are unclear, at least two methods of chromatin decondensation leading to NET formation (NETosis) have been described: NOX2-dependent and NOX2-independent (108). NOX2-dependent NETosis, also termed suicidal NETosis, occurs via activation of NOX2 and production of intra-phagosomal ROS. This causes increased intracellular membrane permeability, movement of elastase to the nucleus and degradation of histones leading to chromatin decondensation and NET release (109). ROS production by NOX2 promotes the morphological changes that occur during NETosis (110) and inactivates caspases to block apoptosis pathways (109). NOX2-independent NETosis does not require the production of ROS by NOX2. In this case, mitochondrial ROS combine with increased intracellular calcium levels to activate peptidyl arginase deiminase (PAD) enzymes (e.g., PAD4) leading to hypercitrullination of histones, chromatin decondensation, and NET release (111, 112). Several inflammatory agents have been reported to induce NET release, including fMLP, IL-8, LPS, nitric oxide, and TNFα (113). Many proteins decorating NET are post-translationally modified, in particular histones which have been found to be methylated, acetylated (114) and citrullinated (111, 115–117) leading to speculation that NETs may be a source of auto-antigens in auto-immune disease (8). Recent work suggests that NETs (MPO:DNA and/or elastase:DNA complexes) detected in up to 79% of RA and up to 100% of SLE sera are generated in a NOX2-independent manner (118).

**Figure 3:**
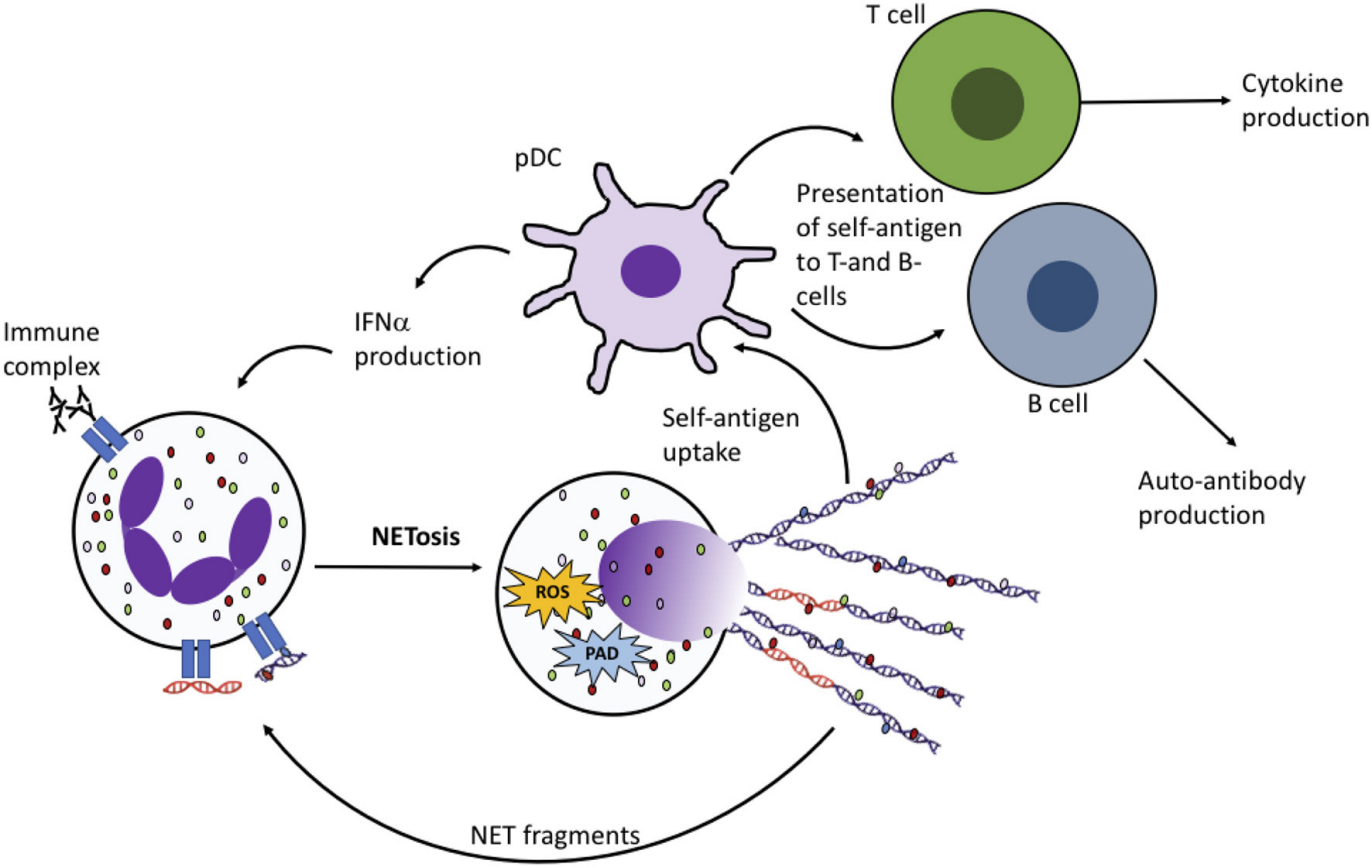
NET production in RA and SLE drives the auto-immune response. Fragments of DNA and proteins (including histones, MPO, elastase, HMGB1, LL37) are taken up by plasmacytoid dendritic cells (pDCs) and presented to auto-reactive B- and T-cells, leading to production of cytokines including interferon alpha (IFNα) and autoantibodies. NET fragments, including oxidized DNA (shown in orange) may also activate NET production by neighboring neutrophils.

There is an ever increasing body of evidence to support the hypothesis that the externalization of double-stranded DNA and post-translationally modified proteins on NETs is implicated in the pathogenesis of SLE, through activation of interferon-producing plasmacytoid dendritic cells (pDCs) (Figure 3) (119) and damage to endothelial tissues and organs (25, 120). SLE sera cross-react *in vitro* with NET components (114, 121) and spontaneous NET production (NETosis) by SLE neutrophils *ex vivo* is also observed (120, 122). NET structures staining positive for DNA, elastase, MPO, and citrullinated histone H3 are found in cutaneous SLE lesions (123) and the kidney (120). Most strikingly the molecular targets of many of the almost 100 auto-antibodies associated with SLE, including those directed at nuclear DNA and nuclear proteins, can be detected in NETs (124). The majority of SLE patients will be positive for antibodies against ANA and/or dsDNA at some point in their disease course (2). Antibodies against histones (including acetylated and/or methylated histones) are also common (125–127). Recent proteomics analysis of NETs from SLE patients identified a number of histone proteins with acetylated, methylated and/or citrullinated residues (128), particularly in NOX2-independent NETs. A number of these, for example acetylated histone H2B (K21, K20), methylated histone H3 (K27) and acetylated histone H4 (K5, K8, K12, K16), correspond to known SLE auto-antibodies (114, 121, 129) and serum NET debris (130). Aside from histones, a number of rarer SLE auto-antibodies correspond to proteins identified on SLE NETs (128) including HMG-17 (131), catalase (132), lamin B1 and B2 (133–135), apolipoprotein A1 (136), cathelicidin (LL37) (137–139), annexin AI and α-enolase (140, 141). When directly compared with RA NETs, the levels of MPO, leukocyte elastase inhibitor and thymidine phosphorylase were higher in SLE NOX2-dependent NETs, whereas histones H1.0, H2B (type 1-J), H2B (type 2-F), and H4 were higher in SLE NOX2-independent NETs (128).

Many NET proteins also correlate with SLE disease activity and play a direct role in tissue damage. MMP9 contained within SLE NETs activates endothelial MMP2, inducing endothelial dysfunction and apoptosis (142). Neutrophil gelatinase-associated lipocalin (NGAL) is both a urinary biomarker of lupus nephritis and a predictor of disease flare, although it is not known whether degranulating neutrophils or NETs are the source of NGAL in SLE urine (143–145). Auto-antibodies to NET proteins that become deposited within the glomeruli of patients with lupus nephritis (141) attract complement and leukocytes expressing Fcγ and complement receptors (including other neutrophils), activating the infiltrating cells causing further tissue damage, e.g., via ROS and protease degranulation (86, 146). In addition, excess NET production within the vasculature and glomeruli promotes vascular leakage and endothelial-to-mesenchymal transition (EndMT), a process that is associated with pathogenic fibrosis of tissues (147). NET production via PAD2 and PAD4 has also been shown to contribute to the development of atherosclerosis and vascular stiffness in murine lupus models (148, 149). Auto-antibodies against apolipoprotein A1 may neutralize the cardioprotective effect of the HDL complex, causing cardiovascular disease in SLE patients with anti-apolipoprotein A1 antibodies (136, 150). Whilst cathelicidin/LL37 may not play a direct role in tissue damage in SLE, its indirect roles include activation of type-I interferon production, activation of the inflammasome and further activation of NET production (137). The abundance of NET debris in SLE sera may be due to impairments in clearance mechanisms associated with tissue homeostasis. Both inhibitors of DNase-I, and anti-NET antibodies in SLE serum, have been proposed as mechanisms impairing the dismantling of NET structures. Impairment of DNase-I is also associated with kidney involvement in SLE (151).

Immune complexes in the sera of SLE patients may contain nucleic acids (Sm RNP RNA or DNA), which activate neutrophils to produce ROS and IL-8 via FcγR2a (152). This phenomenon is inhibited by chloroquine, a DMARD commonly used to treat SLE. SLE immune complexes often contain LL37:DNA complexes derived from NETs. LL-37:DNA complexes activate B cells via TLR9 leading to expansion of self-reactive memory B cells and IgG production (153). B cell activation by LL37:DNA can also be inhibited by chloroquine, suggesting an important role for endosomal TLR activation by NETs in the pathology of SLE. In a recent clinical study of 16 SLE patients, excessive NET production by was abrogated by combination therapy with rituximab (a B cell targeted therapy) and belimumab (anti-BLyS/BAFF) (154). Low disease activity, including a significant reduction in auto-antibody titers, was achieved in over 50% of patients in the study. Further analysis of sera from the patients in this study revealed that the combination of rituximab and belimumab significantly decreased anti-dsDNA, anti-histones, anti-nucleosomes and anti-C1q titers (155). In addition, the ability of patient sera to induce NET formation *in vitro* was decreased by around 75% following combination therapy with rituximab and belimumab (155).

In RA it is the exposure of citrullinated proteins on NETs that is a key driver of auto-immunity, leading to the development of anti-citrullinated peptide auto-antibodies (ACPA) (8, 156). NET debris can be detected in both serum and synovial fluid from RA patients (98, 118) and NET structures staining positive for CD15, elastase, MPO and citrullinated (cit) histone H3 can be seen in synovial biopsy tissues (156, 157). Many proteins in RA patient neutrophils are citrullinated via activation of PAD2 and PAD4 (158), including known auto-antibody targets: cit-actin, cit-histone H1.3, cit-histone H3, cit-vimentin (156, 158). PAD enzymes are present in synovial biopsies, localized with MPO in necrotic areas of synovial tissue that also contain large areas of citrullinated proteins (159), and PAD2 and PAD4 are present in NETs generated from *ex vivo* RA neutrophils (128). PADs are also found in high concentrations in synovial fluid alongside citrullinated proteins, such as α-enolase (160, 161). Proteomics analysis of RA NETs identified citrullinated forms of known auto-antibody targets, such as cit-α-enolase, cit-histone H2A, cit-histone H4, cit-vimentin (128), as well as acetylated and methylated histones in line with analysis of NET debris in RA serum (118). Proteomic analysis of synovial fluid from RA and spondyloarthritis (SpA) patients identified many neutrophil proteins present at significantly elevated concentrations in RA synovial fluid, including MPO, cathepsin G, annexin-A1, and NGAL. Although no difference was observed in the amount of cell-free DNA between RA and SpA synovial fluid, the levels of 21 NET proteins were elevated in RA SF, including histones H2A, H2B and H4, MMP9, elastase, and α-enolase (98). Whilst the levels of ACPA in RA serum do not appear to correlate with NET material, antibodies to NET material (ANETA) are significantly higher in seropositive RA patients (162).

Several mechanisms have been proposed explaining how NETs and NET proteins contribute to joint damage and disease activity in RA. Elastase within in NETs has been demonstrated to disrupt the cartilage matrix, triggering PAD2 release from fibroblast-like synoviocytes (FLS). Activated PAD2 causes citrullination of cartilage fragments which are then internalized by FLS and presented via MHC Class II to antigen specific T-cells leading to the production of ACPA in HLA-DRB1^*^04:01 transgenic mice (163, 164). Both MMP8 and MMP9, found in RA NETs, contribute to degradation of the cartilage matrix and are associated with increased mortality (100, 165, 166). NETs also contain enzymes which degrade aggrecan, another key structural component of cartilage (164). Citrullinated vimentin and aggrecan are preferentially recognized by T cells expressing the HLA-DRB1^*^04:01/04 allele (known as the “shared epitope”) (163, 167) inducing auto-antibody production (164). Auto-antibodies secreted by RA synovial B cells cross react with cit-fibrinogen, cit-histones H2A/H2B, and cit-vimentin, as well as NETs generated from RA blood and joint neutrophils (168). Presentation of citrullinated antigens to autoreactive T cells provides a molecular explanation for the strong association between the HLA-DRB1*04:01/04 allele and the development of RA (164, 167).

## Gene Expression and Cell Signalling

For many years, mature neutrophils were wrongly believed to be transcriptionally silent, and any changes in protein levels during activation were believed to be solely due to mobilization of internal stores during priming and/or membrane shedding rather than synthesis of new protein. However, there is now an increasing body of work demonstrating that neutrophil gene expression is dynamic, being rapidly regulated over short time points by exposure to inflammatory agents, such as TNFα, GM-CSF, IL-1β, LPS, and opsonized micro-particles (169–171), and over several hours by chromatin remodeling, for example in response to the TLR8 agonist resiquimod (R848) (172). Neutrophils have the capacity to express and secrete a wide range of inflammatory mediators, including interleukins (including IL-1α and−1β, IL-1RA, IL-6, IL-12, and IL-23), chemokines (CCL and CXCL family members), TNF superfamily members (including TNFα, BLyS/BAFF, APRIL, TRAIL, and RANKL), metabolites of arachidonic acid (leukotriene B4, prostaglandin E2, and thromboxane A2), and angiogenic factors, such as VEGF and HGF (8, 22, 173–177).

Neutrophil gene expression is highly regulated during granulopoiesis (178–180), with transcripts for granule proteins (e.g., MPO, elastase, lactoferrin) becoming depleted once the mature protein has been packaged within granules in the maturing neutrophil (181). The content of neutrophil granules is determined by the order in which the granules develop: azurophilic granules first, followed by specific granules, then gelatinase granules, and finally secretory vesicles (182). Expression of neutrophil receptors (integrins, FcγR, cytokine receptors) is also highly controlled during granulopoiesis, with neutrophil markers CD16 (FcγR3b) and CD10 (neprilysin) for example only being expressed as the mature neutrophil is ready to exit the bone marrow (181, 183). Many neutrophil genes, including those coding for granule proteins, migratory proteins, chemokines, and chemokine receptors are also regulated by circadian rhythms (19). Dynamic changes in neutrophil gene expression also take place as cells migrate from the blood into inflamed tissues during inflammation, with transcripts for adhesion molecules decreasing and chemokine expression increasing at sites of inflammation (49, 184). Polymorphisms within expression quantitative trait loci (eQTLs) have been found in over 160 genes which play a fundamental role in every stage of neutrophil biology, from granulopoiesis to activation during infection and ultimately apoptosis. Several of these eQTLs are associated with known Mendelian disorders and inflammatory diseases (185).

Transcriptomic analysis of RA and SLE neutrophils has revealed a strong IFNα-induced gene expression signature in both conditions (186, 187). In RA, a high IFNα-regulated gene expression signature is a predictor of response to TNF inhibitor therapy (188). IFNα has recently been shown to regulate neutrophil activation in the presence of inflammatory cytokines (173) and in particular is involved in a switch in expression of chemokine genes. Whilst expression of genes for *CCL* and *CXCL* family chemokines is relatively low in blood neutrophils from RA patients with a high interferon gene expression signature, the expression of these chemokines increases significantly in RA synovial fluid neutrophils following migration to inflamed joints (49, 189–191). RA blood neutrophils also express mRNA for MHC Class II, with both RNA and MHC Class II protein being detected in RA synovial fluid neutrophils (192). Together this activated, synovial fluid neutrophil phenotype is responsible for driving activation of innate and adaptive immune cells in RA tissues. A polymorphism in the leukocyte phosphatase PTPN22 (R620W, rs2476601) enhances migration of RA neutrophils, and causes increased production of ROS following TNF-priming *in vitro* (193). Neutrophils from RA patients also express significantly higher levels of membrane proteinase-3 (mPR3) compared to healthy controls, an observation that is found in other neutrophil-driven autoimmune diseases, such as vasculitis, but not in T-cell driven type-I diabetes (194). In some RA patients, mRNA levels of granule genes (e.g., elastase, MPO) remain elevated even in mature blood neutrophils despite these transcripts normally being down-regulated during granulopoiesis (188). Expression of neutrophil granule genes is associated with non-response to TNF inhibitor therapy in RA but does not correlate with intracellular levels of granule proteins (188). Expression of membrane TNFα correlates with DAS28 and is decreased following successful treatment with TNF inhibitor therapy (48).

A global down-regulation of miRNA expression has been shown in RA blood and synovial fluid neutrophils compared to healthy controls (191). Decreased miRNA levels correlate with clinical parameters of disease including ACPA titer and DAS28 scores and is more pronounced in synovial fluid neutrophils. Targets of these down-regulated miRNAs include genes associated with cell migration, cell survival and inflammation, suggesting that a defect in miRNA expression may drive neutrophil-mediated inflammation within RA joints (191). An eQTL in the gene encoding PAD4 (*PADI4*, rs2240335-A) is associated with increased expression of PAD4 in neutrophils and is in almost complete linkage disequilibrium with the rs230188 SNP in *PADI4* (LD *r*^2^ = 0.93) that confers an elevated risk of developing RA (185, 195).

In SLE, the IFNα-induced gene expression signature is believed to result from activation of pDCs by nuclear debris and NET fragments. The IFNα-induced gene expression signature can be detected in neutrophils, PBMCs and whole blood transcriptome analysis (187, 196–198), and in some studies has been shown to correlate with organ involvement and/or disease activity (198). However, a subset of SLE patients do not display this signature and indeed SLE patients are often described as being interferon “high” or “low” (196, 199). SLE neutrophil DNA is hypomethylated, especially near interferon-response genes (200). Some success in stratifying interferon-high patients to anti-IFNα therapy (anifrolumab) is evident (201), although the largest clinical trials of anifrolumab (TULIP-1 and TULIP-2) found no significant differences in the response to anti-interferon therapy between the interferon-high and interferon-low patient groups (202, 203). Expression of neutrophil genes within SLE whole blood has been attributed to the increase in the population of low density granulocytes (LDGs), discussed below (198), although proteomics analysis of SLE neutrophils and LDGs suggests that protein levels of MPO and other granule proteins is higher in SLE neutrophils (204). The expression of neutrophil granule protein genes in SLE whole blood is strongly associated with lupus nephritis (197, 205), vascular inflammation and cardiovascular involvement (206). SLE neutrophils also express high levels of BAFF/BLyS (198), the molecular target of belimumab which is the first biologic therapy developed to specifically target SLE (207).

## Immunometabolism

Cellular metabolism is key regulator of neutrophil energy production, activation and function under conditions of both homeostasis and inflammation. The complex interplay of metabolic pathways and the utilization of shared metabolic intermediates has been the subject of great focus in immunology research as well as in diseases of metabolic dysfunction and aging (208–210). Complex changes in metabolic regulation within leukocytes occur during activation, when migration from blood to tissue or differentiation into tissue resident immune cells causes dramatic changes in nutrient and oxygen availability, placing sudden and high metabolic demands upon immune cells (211, 212). Fine-tuning of metabolism during an inflammatory response is a key for the generation of small molecule metabolites, such as ATP, NADPH, nucleotides, and amino acids, which are required rapidly and in high abundance during cellular activation (211, 212). Dysregulation of metabolic control has been described in inflammatory diseases, such as RA (213), where changes in T-cell glycolytic activity drives differentiation, hyperproliferation, and hypermigration of T-cell subsets (213–215).

**Figure 4:**
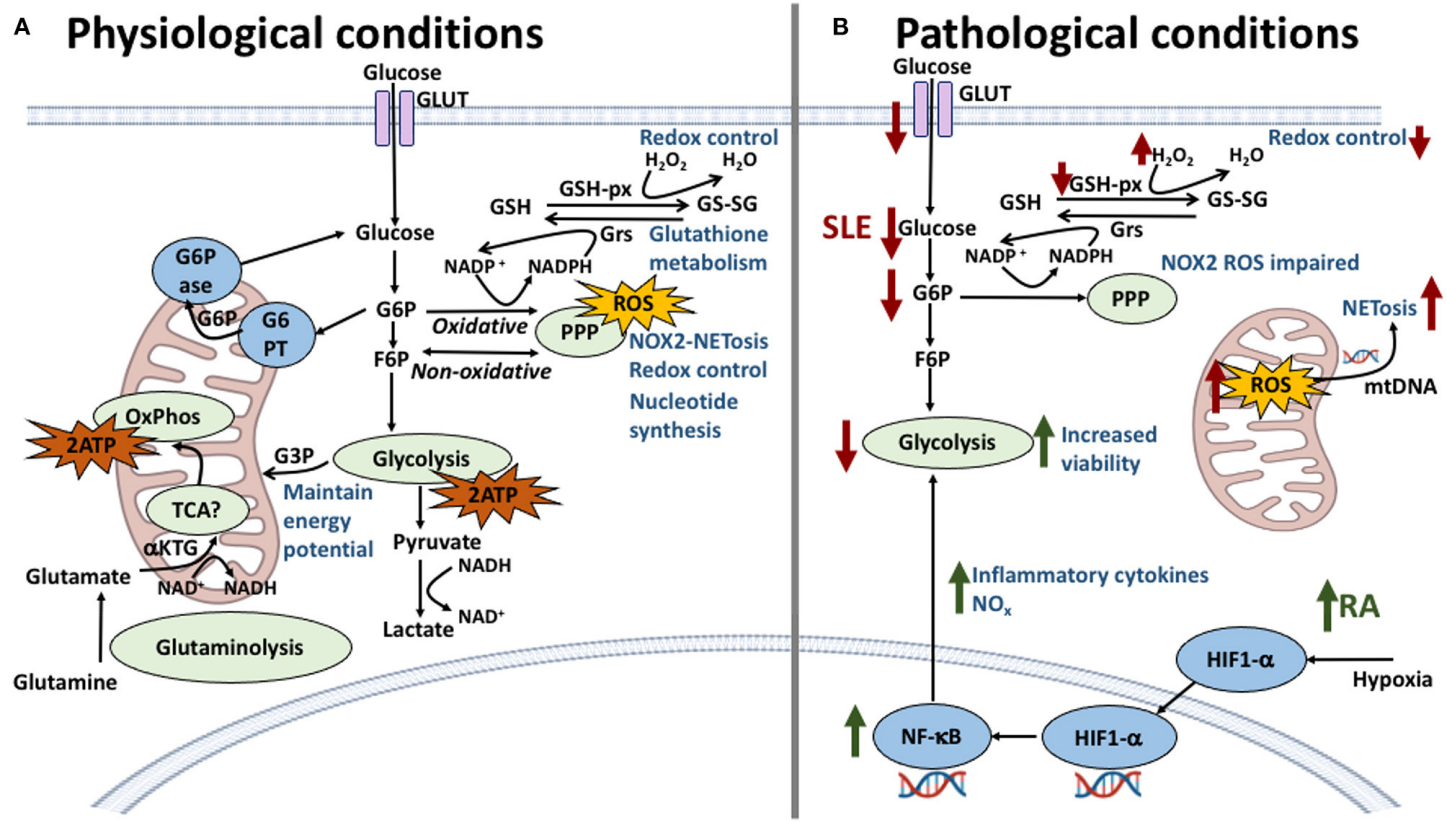
Neutrophil metabolic pathways and the effect of their dysregulation in SLE and RA. **(A)** In physiological conditions, glycolysis is the main energy producing pathway utilized by neutrophils; intermediate metabolites G6P and F6P are starter molecules for the pentose phosphate pathway (PPP), important in redox control, NOX2-dependent NETosis and ROS production. The oxidative stage of PPP produces NADPH which is used in the glutathione metabolism to reduce glutathione providing further redox capacity. Other energy producing pathways have been described, previously thought to be non-functional, such as the TCA cycle which connects glutaminolysis pathway and further regulates NOX2 complex by generating the reducing equivalents of NADPH. **(B)** In SLE and RA dysregulation of these pathways is responsible for the conditions observed at the cellular level. In SLE (red arrows), a lower expression of glucose transporters is met with lower levels of intracellular glucose, diminishing the energy output by glycolysis and compromising cellular viability. Furthermore, a decrease of G6P intermediates correlates with lower levels of NOX2-dependent ROS and decreased redox capacity. NOX2-independent ROS production in mitochondria is amplified in SLE neutrophils with increasing release of NETs containing mitochondrial DNA. In RA (green arrows), the inflammatory environment and hypoxic conditions increase the expression of HIF1-α which upregulates key glycolytic enzymes increasing energy production and viability. HIF1-α also is an upstream regulator of NF-κB which increases pro-inflammatory cytokine production therefore maintaining the inflammatory environment.

Glucose metabolism has its greatest role in neutrophil energy production (Figure 4); neutrophils are known to rely on glycolysis to fuel their energy requirements (216), where the multi-step enzymatic conversion of glucose into pyruvate in the cytosol provides relatively low levels of ATP and NADH. Pyruvate would normally be oxidized by mitochondria through the tricarboxylic acid (TCA) cycle in aerobic conditions, however in neutrophils it is converted instead into lactate, enabling the generation of NAD^+^ for re-use in the glycolytic pathway. The first intermediate of glycolysis, glucose 6-phosphate (G6P), fuels the pentose phosphate pathway (PPP). NAPDH is produced in the oxidative phase of the PPP, maintaining NOX2 activity and ROS production, and is necessary for chromatin decondensation, NOX2-dependent NET formation and NET release (217). The non-oxidative step of the PPP generates nucleic acids and glycolytic precursors. Tight control over glucose metabolism and PPP activity is achieved by the glucose-6-phosphate transporter (G6PT)/G6Pase-β complex which maintains cellular energy homeostasis and functionality in neutrophils by limiting G6P availability in the cytoplasm. Dysfunction in glucose homeostasis due to defective G6PT activity in neutrophils impairs ROS production, calcium mobilization and chemotaxis (218, 219).

A number of other metabolic pathways have been described in neutrophils, including the Krebs/TCA cycle, oxidative phosphorylation (OXPHOS), and a fatty acid oxidation (FAO) pathway, all taking place within the mitochondria. Early experiments suggested that neutrophil mitochondria were not fully functionally active, having a dispensable role in neutrophil respiration and energy production. Mitochondrial density in human neutrophils is low and inhibitors of OXPHOS do not alter the rates of oxygen consumption or H_2_O_2_ production (220–222). However, the notion of metabolically inactive mitochondria was recently challenged by a study in mice which suggested that a metabolic shift from glycolysis to OXPHOS for energy sustainability was possible in neutrophils, both during changes that occur within the cancer tumor microenvironment, and in a number of neutrophil subsets (223). Mitochondria also have a wider and more important role in the regulation of aerobic glycolysis by maintaining the energy potential through complex III of the respiratory chain. Glycerol-3-phosphate (G3P), a by-product of glycolysis, can enter mitochondria where it is re-oxidized on the outer surface of the inner membrane, effectively maintaining membrane potential (224, 225). A further role for neutrophil mitochondrial metabolism in neutrophil function has been demonstrated, with mitochondrial activity being central to processes including ROS production, chemotaxis, and apoptosis (226–230). There is also increasing evidence of the utilization of the TCA cycle by neutrophils for energy metabolism under glucose-limited conditions (231) and during granulopoiesis (232). In addition, LDGs convert glutamate or proline to alpha-ketoglutarate (α-KG) to feed the TCA cycle and enable them to perform metabolically demanding neutrophil functions under conditions of glucose deprivation (233).

Parallel to glucose and mitochondrial metabolism, which are restricted to the cytosol and the mitochondria, glutaminolysis is a metabolic pathway that encompasses both environments and is tightly correlated with the phagocytic and bacterial killing ability of cultured neutrophils. Interestingly, neutrophils have been found to utilize more glutamine than other leukocytes including lymphocytes and macrophages (234). The precursor glutamine is not completely oxidized to generate ATP, and instead plays a role in the regulation and activation of the NOX2 complex, joining glucose metabolism in providing the means of rapidly generating the reducing equivalents of NADPH for the microbicidal NADPH oxidase (NOX2) system (235, 236). Glutaminolysis converts glutamine into TCA cycle metabolites, linking the two pathways together. Glutamine goes through a series of biochemical reactions where it is initially converted to glutamate, and then into α-ketoglutarate, which oxidizes NAD^+^ into NADH. At this point α-ketoglutarate enters the TCA cycle and is converted to malate which is then transported out of the mitochondria to be converted into pyruvate, which just like in glucose metabolism is converted into lactate to generate NAD^+^ (234, 237). Under conditions when glucose supply is limited, neutrophils can switch to the utilization of glutamine to meet their energetic needs (238). Furthermore, glutamine addition to cell culture medium of isolated neutrophils increases their phagocytic activity and rate of superoxide production (239). Glutamine has also been found to indirectly affect the functionality of neutrophils by modulating the production of IL-8, a neutrophil chemoattractant, in various cell types during activation (240, 241).

Within RA synovial fluid, a dynamic environment exists that controls neutrophil fate by a variety of anti- and pro-apoptotic factors. Low oxygen levels in the RA joint trigger a delay in apoptosis (58). HIF1-α is upregulated in hypoxic conditions by the family of oxygen sensing proteins known as prolyl hydroxylase domain enzymes (PHD1-3). HIF1-α exerts control over glycolysis by regulating the expression of key glycolytic enzymes, G3PDH and triosephosphate isomerse-1, providing a mechanism for the continued generation of ATP. This mechanism is an essential requirement for neutrophil functional responses to inflammation (242–244) and, by additionally directing the expression of the leukocyte β2 integrin CD18, it is critical for innate and adaptive immune responses (245). Studies have revealed that NF-κB is an important downstream effector of the HIF1-α-dependent response to hypoxia and that knockdown of the HIF1-α gene decreases glycolytic metabolism and induces cell death (59). RA synovial fluid contains significantly lower levels of glucose than OA synovial fluid, reflecting the high levels of cellular metabolism by infiltrating leukocytes and synovial tissues (246). Other metabolites within RA synovial fluid, including citrate, itaconate, and succinate, have been shown to regulate ROS production, cytokine expression and inflammasome activation in leukocytes (247–249), although the direct effect of these individual metabolites on synovial neutrophil function remains to be determined.

The abundance of apoptotic neutrophils in the blood along with their defective clearance and the propensity to form NETs contribute to the pathogenesis of SLE. Consistent studies reporting increased apoptosis, inflammatory phenotypes and mitochondrial defects in SLE neutrophils suggest that their cellular metabolism might be highly skewed (218, 250). SLE neutrophils have been found to have a decreased capacity for glucose uptake via defective expression of glucose transporters (GLUT-3 and GLUT-6) on the cell membrane compared to healthy neutrophils (250, 251). Limited glucose availability may pose a threat to cellular viability due to decreased glycolytic flux. This is true for other immune cells, such as lymphocytes, which enter BCL-2-regulated apoptosis when glycolytic flux decreases to levels that no longer sustain viability (252). Glucose availability is essential for the two primary pathways in activated neutrophils (glycolysis and PPP), and impaired glycolytic flux could explain the impaired NOX2-dependent ROS production seen in some SLE neutrophils, where glucose would be prioritized for energy production over ROS/NET production. Indeed, impairment of the G6PD/glucose flux in experimental models is directly associated with less NOX2 activity and with lower ROS production (253). Decreased levels of NOX2 ROS production may be compensated for by hyper-functional mitochondrial ROS production, which causes oxidation of unprotected mitochondrial DNA which is then extruded through NETosis (122). Increased mitochondrial ROS production and higher levels of O_2_^-^, H_2_O_2_, and HO^•^ in SLE neutrophils (76, 77) are met by a decreased redox capacity compared to healthy controls (68, 251, 254). This is due to lower concentrations of glutathione which is critical for adequate redox capacity (255, 256). Neutrophils have a large capacity to keep glutathione in the reduced form via the activity of glutathione reductase (GRs) (257). A deficiency in this enzyme produces a more transient oxidative burst in response to bacteria (258). On the other hand, age-related impairment of glutathione peroxidase activity accounts for increased intracellular accumulation of hydrogen peroxide (259). Decreased levels of intracellular glutathione peroxidase (GSH-px), due to impaired GRs activity, leads to neutrophil dysfunction during conditions that are associated with chronic inflammation (260, 261). NOX2-independent ROS production in neutrophils is amplified by reagents affecting glutathione homeostasis (262). The wider impact of circulating SLE neutrophils in the modulation of other leukocyte activation is emphasized by the increased release of NETs containing mitochondrial DNA when compared to healthy donors; this was linked to chronic activation of pDCs and amplification of IFNα production (119, 263).

## Neutrophil Subsets

The peripheral blood population of neutrophils is not a homogeneous pool (264); several populations of neutrophils have been identified which circulate alongside mature neutrophils, including low-density granulocytes (LDGs, CD15^high^/CD14^low^/CD10^+/−^/CD16^+^), granulocytic myeloid-derived suppressor cells (G-MDSCs, CD11c^high^/CD62L^low^/CD11b^high^/CD16^high^/CD33^low^), and reverse migrated neutrophils (RM, CD54^+^/CD18^high^/CXCR1^low^) (265– 267). Tumor associated neutrophils (TANs), which may have a pro- or anti-tumor phenotype, are present in many cancers (266, 268, 269); their function is outside the scope of this review.

G-MDSCs are an immune regulatory neutrophil subtype that inhibit the activation and expansion of autologous T cells, via the production of ROS at the immune synapse (270). Expression of MAC-1 (integrin α_M_β_2_) is essential to the suppressor function of G-MDSCs (270). G-MDSCs with the ability to inhibit T cell proliferation are present in RA blood and synovial fluid (271, 272) and have been studied to greater effect in murine models of auto-immune arthritis, where they have been shown to inhibit both T cell proliferation and differentiation of Th1 and Th17 cells, and promote Treg numbers (273–275). G-MDSCs are found at a higher proportion in the blood of SLE patients than in healthy controls (272). SLE G-MDSCs produce high levels of ROS and also have the ability to impair T cell expansion (276). G-MDSCs from lupus-prone mice produce more ROS and NETs than healthy mice, however the population of G-MDSCs is unable to expand under inflammatory conditions, suggesting the loss of G-MDSCs due to NETosis may contribute to the impaired resolution of inflammation in SLE (277).

Reverse migration of neutrophils from sites of inflammation back into the circulation has been observed in zebrafish, mice and humans (267). Zebrafish RM neutrophils remain functional and able to respond to a second inflammatory challenge (278). RM neutrophils express high levels of ICAM-1 (CD54) (279), and in mice have been shown to contribute to systemic inflammation (280). RM neutrophils represent around 1–2% of circulating blood neutrophils in RA patients and only around 0.25% of blood neutrophils in healthy individuals (279). RM RA neutrophils have lower levels of constitutive apoptosis and produce higher amounts of ROS than circulating blood neutrophils (279).

Low-density granulocytes (LDGs) were first reported in the blood of SLE patients in 1986 (281) but their function and pathological significance has only recently been explored. These cells, remaining in the peripheral blood mononuclear cell (PBMC) layer after density-gradient centrifugation, express cell-surface markers specific to mature neutrophils (CD15^high^/CD14^low^/CD10^+/−^/CD16^+^) (282, 283), whilst expressing mRNA transcripts characteristic of immature neutrophils (e.g., MPO, elastase) (120, 283, 284). High numbers of LDGs in SLE blood correlate with skin involvement, vasculitis, dsDNA titers and SLEDAI scores (120, 284, 285), and SLE LDGs have an increased tendency to form NETs *in vitro* (120). Un-stimulated SLE LDGs secrete increased amounts of IL-8 and IL-6 and have impaired phagocytic capacity (282). SLE LDGs also stimulate production of TNFα, TNFβ, and IFNγ by T cells (286). Recent work has revealed that the SLE LDG population is heterogeneous (mature CD10^+^ or immature CD10^−^), with significant differences in transcriptomic and epigenomic regulation of function and phenotype that correlates with clinical manifestations of the disease (187, 284). Mature CD10^+^ SLE LDGs express high amounts of mRNA and protein for interferon-regulated genes, whereas immature CD10^−^ SLE LDGs express high amounts of mRNA for cell cycle genes (187, 204). CD10^+^ SLE LDGs undergo phagocytosis, chemotaxis and NETosis at higher levels than CD10^−^ LDGs, which release MPO at higher amounts than CD10^+^ LDGs and normal density SLE neutrophils (187). It has been suggested that SLE LDGs undergo NETosis in response to the production of mtROS, with SLE LDG NETs containing mitochondrial DNA including oxidized DNA (8-oxo-2'-deoxyguanosine) which is strongly interferogenic (122). This phenomenon is also observed in chronic granulomatous disease LDGs, which lack functional NOX2 but can produce mtROS and NETs (122). SLE LDGs have stiffer biomechanical properties and are slower to migrate through microvascular mimetics *in vitro* (204). This may explain why LDGs are not found in affected tissues in SLE (284).

RA LDGs have a distinct transcriptome profile compared to RA neutrophils, expressing high levels of transcripts for granule proteins and cell cycle checkpoint genes, and lower levels of expression of apoptotic genes, cytokines, chemokines, and signaling receptors (283). The presence of LDGs in RA blood is unaffected by therapy, and LDG counts correlate with measures of disease activity (DAS28) (283). RA LDGs undergo lower levels of apoptosis *in vitro* after overnight culture; however whilst LDG apoptosis can be further delayed by GM-CSF, LDG apoptosis is unaffected by TNF-α. In addition, ROS production by TNFα-primed RA LDGs is lower than paired blood neutrophils, likely due to their lower expression of TNF-receptors (283). NET production by RA LDGs is not significantly different from paired neutrophils (283).

Whilst the main focus of investigation into the phenotype of LDGs has focused on SLE and to a lesser extent RA, their presence in the blood is not exclusive to rheumatic disease. LDGs have been identified in many other disease settings, including asthma, vasculitis, multiple sclerosis and chronic kidney disease (287–290). Indeed, they are even present in low numbers in healthy controls (291). Isolation of LDGs from blood is highly dependent upon the density of the isolation medium used (e.g., Ficoll, Percoll, Polymorphprep) (292) and this raises the question as to whether studies using different isolation protocols for preparation of neutrophils and LDGs from whole blood can be directly compared. There are mixed reports on the functionality of LDGs from healthy controls, and whether they have different immunological properties (e.g., T cell suppression) to normal density neutrophils and LDGs from inflammatory disease (291, 292). Another key question is whether LDGs represent a novel subset of neutrophils, or whether their phenotype reflects one of spectra of phenotypes that blood neutrophils may exhibit through functional plasticity. Evidence from RA and SLE (CD10^−^ LDGs), where LDGs express cell cycle genes and transcripts for neutrophil granule proteins, suggests that these cells may have arisen from emergency granulopoiesis due to chronic inflammation (187, 283). Administration of LPS *in vivo* to healthy volunteers appears to support this conclusion, with an increase in immature CD16^dim^ band cells being observed in blood 3h after LPS challenge (292). However, it is also possible to induce a low density phenotype from normal density neutrophils *in vitro* by activation with agents, such as fMLP, platelet activating factor, TNFα and LPS (291, 292), suggesting that LDGs may represent a subset of primed or activated neutrophils (292). Further work needs to be carried out to determine the true origin, phenotype and nature of LDGs and other neutrophil subsets, such as G-MDSCs (264).

## Neutrophils as a Therapeutic Target

In this review we have discussed the multitude of ways that inflammatory neutrophils drive inflammation in RA and SLE. This raises the potential to target dysregulated neutrophil activation with therapeutics in both diseases (293). In RA, neutrophil activation can be targeted by biologic DMARDs (bDMARDs), such as anti-TNF therapy, which has been demonstrated to decrease neutrophil membrane TNF expression and NF-κB activation (48). Newer orally available, small molecule therapies, such as JAK inhibitors have shown good efficacy in RA, as they target intracellular signaling via a number of cytokine receptors, including IFNα, IFNγ, GM-CSF, and IL-6 (294, 295). JAK inhibitors baricitinib (JAK1/2) and tofacitinib (JAK3/1) inhibit cytokine priming in neutrophils and can inhibit RA neutrophil migration and ROS production (296). In SLE, the bDMARD belimumab inhibits the cytokine BLyS/BAFF, a major source of which is activated neutrophils and LDGs (175, 198). Belimumab is one of only two drugs specifically licensed to treat SLE in the UK, the other being hydroxychloroquine.

Hydroxychloroquine, an anti-malarial already widely used to treat both RA and SLE, is a potent modulator of neutrophil function. It has been shown to inhibit neutrophilic inflammation into inflamed kidneys (297), inhibit NET production via inhibition of TLR9 (298), and block ROS and IL-8 production in response to RNA-containing immune complexes (152). Methotrexate, commonly used in both SLE and RA, inhibits cytokine-delayed neutrophil apoptosis, ROS production and leukotriene B4 synthesis (299–301). Glucocorticoids are frequently used in both RA and SLE to control disease flares. Prednisolone for example, rapidly disarms pro-inflammatory neutrophils and inhibit both ROS release and production of pro-inflammatory mediators (14, 302, 303).

Several newer therapies under development or in clinical trial also target neutrophil activation. Major activators of neutrophil production and priming, the colony stimulating factors (CSFs), are exciting targets for treatment of neutrophil-driven inflammatory diseases. Anti-GM-CSF (mavrilimumab) therapy has had success in RA clinical trials (294), and anti-G-CSF therapy is effective in treating murine arthritis, both inhibiting neutrophil migration into joints, and suppressing cytokine production (304). Neutrophil migration into inflammatory murine joints is also significantly decreased by inhibitors of CXCR1/CXCR2, the receptor for CXCL8 (IL-8) (305). This decrease in neutrophil infiltration is mirrored by lower disease activity and TNFα production within the joint (306). Bosutinib, an Abl/Src kinase inhibitor currently used to treat patients with chronic myeloid leukemia, inhibits neutrophil FcγR2A-induced ROS production, recruitment to glomerular capillaries and kidney injury in an immune complex-driven model of kidney disease (85) suggesting this may be a promising therapy to target neutrophilic damage in lupus nephritis.

Excess NET production represents an exciting prospect for therapeutic development, with the potential to break the chain leading to auto-antigen recognition, activation of pDCs, interferon production, auto-antibody production and damage to local tissues, such as cartilage and microvessels within the kidney. As mentioned earlier, a clinical trial of rituximab and belimumab inhibited NET production in SLE, and this was associated with lower auto-antibody titers (including lower anti-dsDNA and anti-histones) and a decrease in disease activity (154, 155). NET production may also be targeted by inhibitors of PAD4, MPO and neutrophil elastase. MPO and elastase inhibitors reduce neutrophil-driven inflammation in animal models of inflammatory disease and human respiratory disease (307, 308). PAD4 is an enzyme that catalyses the conversion of arginine to citrulline (116, 309). It plays an important role in chromatin decondensation during NETosis and is physically associated with the cytosolic subunits of the oxidative burst machinery in a way that regulates assembly of the active NOX2 complex (116, 309). Over the last few years, PAD4 has emerged as a potential therapeutic for the treatment of RA and SLE. Initially irreversible inhibitor compounds, such as F- and Cl-amide were found to inhibit PAD4 (310), and showed efficacy in RA and SLE models through the inhibition of NET production. Cl-amidine prevented development of atherosclerotic plaques, interferon production and immune complex deposition in the kidney in lupus-prone mice (148, 149, 311). However, their poorly understood involvement in this process was the driving force for discovering reversible inhibitors, such as GSK484 and GSK199. GSK484 is the more potent of the two and selectively targets PAD4. It inhibits citrullination in primary neutrophils and NET formation in both mouse and human neutrophils (312, 313). Currently novel agents targeting PAD4 are being developed, which have shown to decrease the levels of circulating NET DNA in serum (313, 314). However, complete inhibition of NET production may block an important neutrophil function that provides protection against infection (315). Indeed, PAD4 knock out mice are highly susceptible to developing systemic inflammation from bacterial keratitis, where NETs normally function to protect the host from infection at the expense of the cornea (316). PAD enzymes also have a key physiological role in regulating gene expression and cellular differentiation, therefore a more targeted approach to PAD/NET inhibition may be required for development of therapeutics (315).

## Final Summary

In this review we have highlighted the way in which dysregulated neutrophil activation can contribute to the development and progression of RA and SLE. In particular, dysregulated apoptosis and NETosis lead to exposure of intracellular post-translationally modified proteins and DNA activating the adaptive immune response (interferon release, auto-antibody production) and inducing damage to tissues either directly or by activating neighboring cells. Neutrophil degranulation and ROS production damage local tissues and contribute to systemic inflammation. Aberrant neutrophil activation in RA and SLE is caused, in part, by a dysregulation of gene expression and metabolism, via different mechanisms specific to each disease. Targeting unwanted neutrophil activation in RA and SLE may be a promising avenue for investigation and may have fewer side effects than the broad-spectrum immunosuppressants often used to treat these life-limiting auto-immune conditions.

## Author Contributions

MF, ZM, and HW wrote, edited, and approved the final version of the manuscript. All authors contributed to the article and approved the submitted version.

## Conflict of Interest

The authors declare that the research was conducted in the absence of any commercial or financial relationships that could be construed as a potential conflict of interest.
